# A phenotype- and potency-gated extracellular vesicle allocation hypothesis for autism

**DOI:** 10.3389/frcha.2026.1942419

**Published:** 2026-08-28

**Authors:** Burak Tatlı, İbrahim Kamer

**Affiliations:** Prof. Dr. Burak Tatlı Private Practice, İstanbul, Türkiye

**Keywords:** autism, endophenotype, extracellular vesicles, neuroinflammation, precision medicine, synaptic plasticity

## Abstract

Autism is a heterogeneous neurodevelopmental condition for which a single extracellular vesicle (EV) product is unlikely to match every biological context. We hypothesized that four development-coded EV candidates should be allocated by convergent phenotype, biomarker, and patient-derived functional response rather than diagnosis alone. EXO2, a cord-blood-plasma EV candidate, was tested only in an immune-inflammatory endophenotype characterized by regression or severe irritability along with objective inflammatory activity, after excluding pain, sleep disorder, epilepsy, infection, and other mimics. EXO3, a dental-pulp mesenchymal-stromal-cell EV candidate, was tested in a synaptic-developmental endophenotype with prominent language impairment or intellectual disability, but only if the patient-derived neurons show improved neurite, synaptic, and network function. EXO1, a Wharton-jelly mesenchymal-stromal-cell EV candidate, was tested in a circuit-plasticity endophenotype dominated by abnormalities in social communication, sensory, and repetitive behaviors using a concordant neuronal-network assay. R-EXO, an umbilical-cord-tissue mesenchymal-stromal-cell EV candidate, remained an exploratory option for a neurovascular-barrier/extracellular matrix endophenotype. Regression, language delay, or behavior alone are not selection biomarkers. Each lot must pass identity, purity, safety, and phase-specific potency gates. Sequential use of EXO2, followed by EXO1, EXO3, or, in exceptional cases, R-EXO is predicted to outperform ungated or simultaneous use when inflammation masks a residual developmental mechanism. This is a falsifiable preclinical framework and does not support clinical administration.

## 1 Introduction/background

Autism is defined by behavioral symptoms but originates from diverse developmental trajectories and biological mechanisms. Language ability, intellectual function, adaptive skills, sensory responses, repetitive behavior, irritability, epilepsy, sleep, and gastrointestinal symptoms vary widely (1, 2). This heterogeneity makes phenotype-only therapeutic assignment hazardous: the same outward behavior can arise from pain, anxiety, sleep deprivation, seizures, communication frustration, or distinct neural-circuit mechanisms.

Developmental regression is also heterogeneous. Some cohorts show immune, adhesion-molecule, or bioenergetic differences between regressive and non-regressive groups, but no validated inflammatory biomarker defines “regressive autism” (3, 4). Therefore, regression cannot justify an anti-inflammatory product. The new loss of language, motor, or adaptive skills requires diagnostic evaluation for epileptic encephalopathy, hearing impairment, Rett or other genetic syndromes, metabolic and neurodegenerative disorders, catatonia, medication effects, and systemic illness before entry into any experimental framework (2).

Preclinical studies have reported that mesenchymal stromal cell (MSC) EVs can alter microglial activation, NF-kB signaling, synaptic proteins, and autism-relevant behavior in BTBR (an inbred mouse strain used as an idiopathic model of autism-relevant behavior), valproate, and maternal-immune-activation mouse models (5–8). A 2026 study also reported the rescue of cellular and behavioral phenotypes in SHANK3-associated neuronal and mouse models (9). These findings establish only the mechanistic plausibility. They do not show that EVs improve language, intelligence, social communication, or behavior in autistic people. A randomized trial of cord-blood cells in 180 children found no overall improvement in socialization, autism symptoms, or vocabulary; cord blood cells are not EVs, but the result cautions against extrapolating from uncontrolled regenerative studies (10).

The hypothesis below treats EXO1, EXO2, EXO3, and R-EXO as development codes. The labels do not establish endosomal biogenesis, purity, potency, or clinical activity. The MISEV2023 principles require source documentation, orthogonal characterization, separation controls, dose–response studies, and functionally relevant potency assays (11).

Endophenotype-based stratification has previously been proposed at a conceptual level in precision-medicine discussions on autism, including immune- and metabolic-biomarker subtyping and multiomic clustering. The present framework differs from these three specific respects. First, it links each clinical endophenotype to a defined, currently available extracellular- vesicle (EV) source product rather than a therapeutic class in general, so that allocation is a testable assignment problem rather than a subtyping proposal. Second, it requires a patient-derived functional rescue assay, not a biomarker association alone, before any candidate is considered matched to an endophenotype (Table 1). Third, the four-candidate structure is stated with explicit *a priori* disqualifying criteria so that failure to separate candidates functionally falsifies the assignment rather than being absorbed into a *post hoc* explanation. Therefore, the contribution of this study is an allocation-and-testing protocol for existing regenerative products, not a new biological subtyping system.

**Table 1:** Proposed four-candidate allocation matrix.

Candidate	Phenotypic entry clue	Required functional gate	Key disqualifier
EXO2	Regression, irritability, or fluctuating function plus objective inflammatory activity	Reduced excessive NF-kB/inflammasome/microglial output with neuronal preservation	Phenotype alone; pro-inflammatory net effect; soluble plasma-protein contamination
EXO3	Prominent language impairment, intellectual disability, or global delay	Improved patient-derived neurite, synaptic, or network development measures	No neuronal rescue; unsafe developmental cargo; claim of IQ/language restoration
EXO1	Social communication, sensory, and repetitive/cognitive-flexibility phenotype	Normalization of network dynamics, E/I balance, and activity-dependent plasticity	Global excitation/sedation; no distinction from EXO3 in head-to-head assays
R-EXO	Exploratory neurovascular-barrier/ECM signature	Restored barrier resistance and physiologic ECM/PNN remodeling	Non-specific matrix degradation; absence of barrier/ECM signature; kidney-only potency

ECM, extracellular matrix; E/I, excitation/inhibition; EV, extracellular vesicle; MSC, mesenchymal stromal cell; PNN, perineuronal net.

## 2 The hypothesis/theory

### 2.1 Central hypothesis

A four-candidate EV panel shows biological activity only when the allocation is gated by three concordant layers: a clinically defined endophenotype, an objective molecular or physiological signature, and a patient-derived cell or organoid assay demonstrating candidate-specific rescue. The predicted mapping was EXO2 for immune-inflammatory dysregulation, EXO3 for synaptic-developmental insufficiency, EXO1 for circuit plasticity dysregulation, and R-EXO for neurovascular-barrier/extracellular matrix (ECM) dysregulation. The clinical phenotype is an entry clue and not a sufficient selector.

### 2.2 EXO2: immune-inflammatory/regressive-irritability endophenotype

EXO2 is a cord blood plasma-derived EV candidate proposed for individuals with regression, marked irritability, or fluctuating function only when objective evidence supports active immune-inflammatory dysregulation. Candidate indicators for research stratification include reproducible cytokine or chemokine patterns, inflammatory PBMC (peripheral blood mononuclear cell) signaling, microglial-response signatures in a patient-derived coculture, and neuroimmune imaging measures. None of these is currently validated as a clinical companion diagnostics.

EXO2 must demonstrate net suppression of excessive NF-kB, inflammasome, and microglial inflammatory output while preserving neuronal viability, host defense competence, and developmental signaling. Cytokine ELISA content is insufficient because the detected proteins may be intravesicular, surface-bound, or coisolated soluble plasma proteins. A lot containing high levels of TNF-alpha or TGF-beta1 is not considered “anti-inflammatory” unless its overall functional effect is resolving rather than pro-inflammatory or immunosuppressive. Regression without a functional inflammatory signature falsifies EXO2 assignment.

None of the candidate indicators listed above were proposed as fixed diagnostic thresholds at this stage. As a starting operational framework for future protocols, “objective inflammatory activity” could reasonably be anchored to standardized, already-used instruments and assays. For example, a validated irritability measure, such as the Aberrant Behavior Checklist irritability subscale combined with a defined elevation in one or more peripheral markers commonly reported in autism neuroimmune studies (e.g., IL-6, IL-1β, TNF-α, or CRP), should be used rather than relying only on clinical impression. The specific cutoffs, panels, and reproducibility requirements for such an anchor remain to be established empirically in Gate 0/Gate 1 testing and are not asserted here as validated criteria.

### 2.3 EXO3: synaptic-developmental/language-intellectual endophenotype

EXO3 is a dental-pulp MSC-EV candidate proposed for a subgroup with prominent receptive/expressive language impairment, intellectual disability, or global developmental delay. Dental-pulp cells are neural crest-derived, and their EVs exhibit neurotrophic and neurite-supporting actions in non-autism models (12, 13). However, language delay and intellectual disability are outcomes, not proof of reversible neurotrophic deficits. Genetic etiologies associated with synaptic or chromatin pathways must be characterized rather than excluded because they may define the relevant model and impose a ceiling on reversibility.

Selection requires patient-derived induced pluripotent stem cell neurons or organoids that show a reproducible EXO3-mediated improvement in neurite complexity, synaptic density, excitation-inhibition balance, network synchrony, or stress resilience. Cargo must be screened for developmental liabilities, including miRNAs or growth signals that promote maladaptive proliferation, aberrant differentiation, or oncogenic pathways. The hypothesis predicts functional plasticity in surviving networks but does not predict an increase in IQ or restoration of previously absent language.

Similarly, “prominent language impairment” and “intellectual disability” should not be operationalized using clinical descriptions alone. A potential starting anchor would combine a standardized adaptive/communication measure (e.g., the Vineland Adaptive Behavior Scales communication domain) with a validated language or cognitive assessment appropriate to the individual's communication level. This should be applied at two time points to confirm a stable rather than a fluctuating deficit before EXO3 candidacy. Similar to the EXO2 anchors mentioned earlier, these are proposed starting points for protocol development rather than as validated selection criteria.

### 2.4 EXO1: circuit-plasticity/social-sensory endophenotype

EXO1 is a Wharton-Jelly MSC-EV candidate proposed for a phenotype characterized by social communication difficulty, sensory dysregulation, restricted/repetitive behavior, or cognitive inflexibility when major inflammatory and metabolic drivers are not supported. Its assigned role is neuromodulatory rather than generically regenerative. A qualifying lot should normalize disease-model network measures without causing global excitation, sedation, or loss of physiological variability. Relevant assays include multielectrode array synchrony, excitatory/inhibitory balance, activity-dependent synaptic plasticity, and microglia–neuron interactions.

The distinction from EXO3 is functional: EXO3 is tested for neurite/synaptic developmental support, whereas EXO1 is tested for the modulation of already formed network dynamics. If both candidates rescue the same assay to the same degree, the source-based distinction is not useful, and the hypothesis should collapse to the better-characterized product.

This also directly affects how the source is used throughout the framework. The tissue source (cord blood, dental pulp, Wharton's jelly, and umbilical cord tissue) is used here only as a manufacturing-batch identifier and a starting hypothesis for candidate assignment, not as a proxy for cargo composition. EV cargo is known to vary within a single source across donors, passages, and isolation methods, and no direct comparative proteomic or transcriptomic datasets exist across these four specific products. Accordingly, the framework's actual selection variable is the functional potency assay in Gate 2 (Section 5.3), not the tissue of origin. A lot that fails its assigned functional rescue is disqualified regardless of source. The hypothesis explicitly allows a “source-agnostic” contingency, in which two differently sourced lots that pass the same functional gate are treated as interchangeable for that endophenotype until a source-dependent difference is demonstrated.

### 2.5 R-EXO: neurovascular-barrier/ECM endophenotype

R-EXO is an umbilical cord tissue MSC-EV candidate originally intended for matrix remodeling. Its relevance to autism is uncertain. The brain extracellular matrix and perineuronal nets regulate critical period plasticity, while endothelial and blood–brain barrier biology may contribute to a subset of neurodevelopmental phenotypes (14, 15). “Antifibrotic” activity in kidney models cannot be transferred to the developing brain, where indiscriminate matrix degradation may be harmful.

A plausible, though currently unconfirmed, mechanistic link would involve MMP/TIMP-mediated regulation of extracellular matrix turnover, integrin-dependent signaling at the neurovascular unit, or modulation of tight-junction protein expression at the blood–brain barrier—pathways already implicated in perineuronal-net remodeling and endothelial barrier biology (14, 15). None of these pathways has been demonstrated specifically for R-EXO, and this paragraph is stated as a testable mechanistic hypothesis rather than as evidence. If Gate 2 testing (Section 5.3) does not demonstrate a reproducible, autism-relevant neurovascular or ECM signal for R-EXO through one of these or comparable pathways, R-EXO should be removed from the framework as overly speculative rather than retained by default.

Therefore, R-EXO should only enter an exploratory barrier/ECM arm after demonstrating restoration, not non-specific disruption, of endothelial barrier resistance, tight-junction organization, and physiologic perineuronal-net turnover in relevant models. It is not a default product of autism. If no reproducible neurovascular/ECM endophenotype or beneficial barrier assay exists, R-EXOs should be excluded from further development for this indication.

## 3 Sequential and multiphase use

This framework rejects the routine coadministration of all four preparations. Simultaneous use would obscure which cargo produced benefit or harm, increase immunological and manufacturing complexity, and prevent causal interpretation. A sequence is justified only when the first measurable biological state may conceal the second state.

### 3.1 EXO2 followed by a residual-phenotype candidate

In the inflammatory endophenotype, EXO2 should be tested first *in vitro* or in an appropriate model. After a predefined resolution signature, lower inflammatory output with preserved neuronal function, the residual phenotype determines the second candidate: EXO3 for persistent neurite/synaptic developmental deficits, EXO1 for persistent network plasticity dysregulation, or, exceptionally, R-EXO for a confirmed barrier/ECM defect. A fixed calendar was not proposed. Failure to reach the resolution gate stops the sequence instead of triggering automatic escalation.

The resolution gate above is not proposed as a fixed calendar rule, but a minimum candidate observation window and a defined resolution criterion are needed to maintain a non-arbitrary sequence. Based on the pharmacodynamic persistence and behavioral testing intervals reported in the cited MSC-EV autism models (5–8), a minimum observation window in the range of 2–4 weeks postexposure in rodent models is proposed as a starting parameter. The resolution gate is operationally defined as a prespecified, assay-specific reduction in the primary inflammatory functional readout (Section 2.2) relative to baseline, sustained across at least two independent measurements, and without a compensatory rise in an alternative inflammatory marker. The exact quantitative threshold cannot be fixed in advance of assay validation and should be defined and justified during Gate 1 potency assay validation (Section 5.2) for each specific assay used, rather than applied as a single universal percentage across different readouts.

### 3.2 Direct single-candidate testing

When inflammatory evidence is absent, EXO3, EXO1, or R-EXO should be tested as a single candidate against the vehicle, mock-isolate, unrelated-EV, and alternative-candidate controls. Experiments should also include reverse order and simultaneous exposure. The hypothesis is supported only if the assigned candidate outperforms competing products in the matching endophenotype and loses this advantage in mismatched models.

## 4 Evaluation of the hypothesis

### 4.1 Supporting observations

The model is supported by convergent, indirect observations: autism contains genetically and biologically diverse subgroups (1); inflammatory and mitochondrial signatures occur in subsets rather than universally (3, 4); MSC-EVs can modify neuroimmune and autism-relevant phenotypes in several animal models (5–8); and source-dependent EV cargo can rescue selected SHANK3-associated cellular phenotypes (9). These findings make stratification more plausible than a one-product-for-all model.

### 4.2 Evidence against and boundary conditions

The principal objection is that none of the four phenotype-to-product mappings has been validated in humans. Peripheral biomarkers may not reflect brain biology; patient-derived neurons may not model whole-brain development; language and behavior measures are influenced by learning, environment, and expectation; and EV biodistribution to the human brain is uncertain. Source labels do not guarantee consistent cargo. The negative overall result of the 180-child cord-blood trial, although not an EV study, illustrates the danger of making broad, unstratified regenerative claims (10).

The framework must also avoid cure-oriented language and normalization as the only valued outcomes. Meaningful endpoints should be individualized and include communication access, adaptive functioning, distress, sleep, safety, participation, and caregiver-identified goals while preserving autonomy and monitoring for loss of valued traits. Established behavioral, educational, communication, and medical support continues regardless of experimental assignment (2).

A further boundary condition, not addressed above, is that the four-candidate panel does not map onto every biologically documented autism subgroup. A mitochondrial-oxidative subgroup—associated in systematic reviews and meta-analyses with developmental regression, seizures, motor delay, and gastrointestinal abnormalities, and distinguishable by biomarkers such as lactate, pyruvate, and mitochondrial DNA copy number—is not assigned to EXO1, EXO2, EXO3, or R-EXO here (16). Nor is there a gut-immune-microbiome axis, where the transplantation of human autism-associated gut microbiota into germ-free mice was sufficient to induce autism-relevant behaviors in one causal experiment, with candidate bacterial metabolites subsequently shown to modulate neuronal excitability (17). Extending the panel to cover these axes, or stating explicitly that they are out of scope for this framework, is a necessary next step for any revision of this hypothesis, not an omission to be resolved silently by implication.

A further boundary condition concerns time. Autism is a dynamic neurodevelopmental process rather than a fixed collection of biological traits, and the molecular and functional signatures used to assign an endophenotype in this framework—inflammatory activity, synaptic-developmental measures, network plasticity indices, and barrier/ECM signals—may themselves change over the course of development, independent of any intervention. A single cross-sectional allocation is, therefore, provisional rather than permanent, and any preclinical or future translational protocol built on this framework should reassess the endophenotype status at defined intervals rather than assuming it is stable.

A related boundary condition is that the four endophenotypes are not presented here as mutually exclusive categories. Inflammatory, synaptic developmental, network plasticity, and neurovascular/ECM mechanisms may coexist within the same individual and may become more or less prominent as development proceeds. The current framework selects a primary candidate based on the dominant, functionally confirmed signature at the time of testing; it does not exclude the possibility that an individual moves between endophenotype assignments over time; and it does not resolve cases in which two or more signatures are functionally confirmed simultaneously. Extending the model to formally handle such overlaps is identified here as a defined direction for revision rather than a silent limitation.

Sex-related biological differences are likewise not yet incorporated as a structural element of the four-endophenotype model itself, beyond the mandatory covariate specified in Gate 0 (Section 5.1). Whether sex should further modify endophenotype boundaries or functional-gate thresholds is an open, testable question rather than an assumption made by this framework.

## 5 Hypothesis testing

### 5.1 Gate 0: clinical and etiologic characterization

Before biological stratification, research participants require standardized autism assessment, developmental and regression history, language, and cognitive testing appropriate to their communication level, adaptive function, sleep, pain, gastrointestinal and psychiatric review, EEG when indicated, hearing evaluation, medication review, and guideline-consistent genetic/metabolic investigation. Acute or treatable causes of deterioration were addressed first. This gate prevents the mislabeling of distress or medical illness as an autism endophenotype.

Because subclinical seizures and metabolic crises can produce a clinical picture resembling inflammatory regression, a minimum diagnostic workup is required before regression is attributed to an immune-inflammatory mechanism for EXO2 candidacy. This should include, at minimum, EEG (sleep-deprived or prolonged if the routine study is non-diagnostic and clinical suspicion remains), a basic metabolic and mitochondrial screen (including lactate, pyruvate, and ammonia), and a review for other treatable causes already listed in Section 1. A regression that resolves or is fully explained by seizure control or metabolic correction does not qualify as an immune-inflammatory endophenotype in this framework.

Sex was recorded as a mandatory covariate at Gate 0 for all four endophenotypes rather than being analyzed *post hoc*. This is warranted because immune-inflammatory, synaptic, and neurovascular barrier biology are each modulated by sex hormones, and autism itself shows marked sex-related differences in prevalence and phenotype. When the sample size permits, endophenotype-specific and sequence-specific analyses should be stratified by sex. If stratification is not possible, sex must be reported, and its exclusion from a given analysis should be explicitly justified rather than left unaddressed.

### 5.2 Gate 1: product identity and safety

Every lot requires documented source, donor, and manufacturing conditions; particle concentration and size; morphology; EV-associated and negative markers; sterility, endotoxin and mycoplasma testing; residual DNA; soluble-protein and lipoprotein assessment; stability; and batch comparability (11). Plasma-derived EXO2 requires stringent separation controls. Tumor-supporting signals, thrombogenicity, complement activation, immune suppression, developmental toxicity, and off-target organ distribution must be evaluated.

### 5.3 Gate 2: endophenotype-specific potency

Blinded head-to-head assays should compare all four candidates using patient-derived immune cells, neurons, glia, endothelial cells, organoids, or microphysiological systems. Potency must be a concentration-dependent, reproducible functional rescue and must outperform mock-processed source material, EV-depleted fractions, and at least one mismatched EV candidate. Particle count, CD9/CD63/CD81 positivity, cytokine abundance, and miRNA lists are characterization data, not potency by themselves.

Given the cost, accessibility, and scalability constraints of patient-derived induced pluripotent stem cell neurons, glia and organoids, an initial screening tier using immortalized neuronal or glial cell lines and commercially available, non-patient-specific organoid models is a reasonable first pass to exclude clearly non-potent or unsafe lots before committing to patient-derived resources. Patient-derived models should be reserved for confirmatory testing of candidates that already pass this surrogate screen. This approach maintains scientific rigor at the confirmatory stage while making the overall gate structure feasible for smaller laboratories.

### 5.4 Gate 3: preclinical sequence testing

Candidate sequences should be tested across complementary genetic, environmental, and patient-derived models. The required comparisons are assigned to a single product, alternative single products, simultaneous exposure, reverse order, and vehicle. Pharmacokinetics, brain and peripheral biodistribution, immune response, seizure threshold, sleep/activity changes, and long-term neurodevelopmental effects are coprimary safety domains. No dose, route, or interval for children can be inferred from rodent intranasal studies.

## 6 Consequences and discussion

If confirmed, this hypothesis would replace diagnosis-based EV selection with a product-agnostic competition among lots and mechanisms. EXO2 would not be “the regression product,” and EXO3 would not be “the speech product.” Instead, regression and speech phenotypes would trigger specific tests, and the product would be selected only after a functional rescue. Non-response would be informative, as it could indicate an incorrect endophenotype, a non-potent lot, irreversible developmental constraints, or a mechanism outside the four-candidate panel.

This point deserves explicit restatement here because it is the hinge of the entire proposal: at every stage of this framework, phenotype and even biomarker positivity serve only as entry clues that authorize functional testing, never as proof that a given mechanism is actually operating in a given individual. A candidate is matched only after a patient-derived functional assay confirms rescue; phenotype alone, however characteristic, is never sufficient grounds for product selection under this framework.

If the four-way mapping fails, the most likely conclusions are that the phenotypes are too coarse, peripheral biomarkers do not capture the relevant brain state, or source-dependent labels add no value beyond a common potency assay (Table 2). Any of these outcomes would refine EV development and reduce empirical exposure. Conversely, positive preclinical findings would justify only a regulated, staged early-phase trial with independent ethics review, assent when possible, parental permission, long-term surveillance, and continued standard support.

**Table 2:** Falsifiable predictions.

Prediction	Supportive result	Refuting or revising result
Matching advantage	Each candidate is superior in its assigned endophenotype	Source label does not perform better than random allocation
EXO2 requirement	Inflammatory functional signature predicts EXO2 response	Regression/irritability alone predicts equally well, or EXO2 worsens inflammation
EXO1 vs. EXO3	Network modulation and neurite-development assays separate the candidates	Both products are functionally indistinguishable
R-EXO boundary	Benefit appears only with a barrier/ECM signature and restorative assay	No reproducible endophenotype or harmful matrix disruption
Sequence effect	EXO2 followed by the residual-phenotype candidate beats either alone and couse	No order effect, reverse order superior, or added toxicity
Clinical boundary	Preclinical signals justify regulated trials only	Uncontrolled clinical use is claimed to be needed to demonstrate the mechanism
Ethical scope boundary (new)	Core social-communication style and identity remain unchanged while the assigned endophenotype's own target measure (inflammatory signature, neurite/synaptic assay, or network-plasticity assay) improves	No endophenotype-specific improvement is observed, or a global, non-specific “improvement” is reported; instead, the latter would itself raise concern for placebo, expectancy, or reporting bias effects rather than validate the hypothesis

No completed controlled trial currently establishes that an exosome product treats autism. The U.S. Food and Drug Administration states that regenerative medicine therapies are not approved for the treatment of autism and that there are no FDA-approved exosome products (18, 19). A recently registered childhood autism EV study did not provide evidence of efficacy (20). Accordingly, this study presents a research hypothesis, not a prescription, compassionate-use rationale, or clinical protocol.

As noted in Section 4.2, this framework does not extend to the mitochondrial-oxidative or gut-immune-microbiome subgroups of autism (16, 17). This omission is a defined direction for future revision, not a silent gap to be inferred by the reader.

If Gate 2 and 3 preclinical testing (Sections 5.3–5.4) supports the framework, the next appropriate step is not clinical administration but a defined early translational program built on multimodal biomarkers rather than any single measure. Such a program would combine clinical/behavioral characterization (Section 5.1), peripheral molecular signatures used in Gate 0/Gate 1 (e.g., cytokine, metabolic, or genetic panels), structural and functional neuroimaging where feasible, and continued patient-derived functional assays as a translational bridge rather than a one-time selection tool. Each modality would need its own validation against the same functional rescue endpoint before it can be trusted as a stand-alone allocation criterion, and none is proposed here as sufficient on its own.

## 7 Conclusion

A phenotype- and potency-gated four-candidate EV framework is testable, but only if the phenotype is treated as an entry clue and not as proof of the mechanism. EXO2 was conditionally assigned to objectively inflammatory states; EXO3 to patient-model-confirmed synaptic developmental deficits; EXO1 to circuit plasticity dysfunction; and R-EXO to an exploratory neurovascular/ECM subgroup. Sequential EXO2-to-residual-phenotype testing is plausible when inflammation precedes the second mechanism, but routine multiproduct use is not. The framework is falsified by the absence of functional separation, lack of sequence advantage, unsafe cargo, or failure of biomarkers to predict response. By its own account, it is also incomplete: mitochondrial-oxidative and gut-immune-microbiome subgroups remain unaddressed and are flagged here as a defined direction for extension rather than an unstated limitation. However, clinical administration remains premature.

## Data Availability

The original contributions presented in this study are included in the article/Supplementary Material, further inquiries can be directed to the corresponding author.
